# Human IgE against the major allergen Bet v 1 – defining an epitope with limited cross-reactivity between different PR-10 family proteins

**DOI:** 10.1111/cea.12230

**Published:** 2014-01-22

**Authors:** M Levin, A M Davies, M Liljekvist, F Carlsson, H J Gould, B J Sutton, M Ohlin

**Affiliations:** 1 Department of Immunotechnology, Lund UniversityLund, Sweden; 2 Randall Division of Cell and Molecular Biophysics, King's College LondonLondon, UK; 3 Medical Research Council & Asthma UK Centre in Allergic Mechanisms of AsthmaLondon, UK

**Keywords:** allergy, antibodies, epitopes, molecular biology

## Abstract

**Background:**

The interaction between IgE and allergen is a key event at the initiation of an allergic response, and its characteristics have substantial effects on the clinical manifestation. Despite this, the molecular details of the interaction between human IgE and the major birch allergen Bet v 1, one of the most potent tree allergens, still remain poorly investigated.

**Objective:**

To isolate Bet v 1-specific human monoclonal IgE and characterize their interaction with the allergen.

**Methods:**

Recombinant human IgE were isolated from a combinatorial antibody fragment library and their interaction with Bet v 1 assessed using various immunological assays. The structure of one such IgE in the single-chain fragment variable format was determined using X-ray crystallography.

**Results:**

We present four novel Bet v 1-specific IgE, for one of which we solve the structure, all with their genetic origin in the IGHV5 germline gene, and demonstrate that they target two non-overlapping epitopes on the surface of Bet v 1, thereby fulfilling the basic criteria for FcεRI cross-linkage. We further define these epitopes and for one epitope pinpoint single amino acid residues important for the interaction with human IgE. This provides a potential explanation, at the molecular level, for the differences in recognition of isoforms of Bet v 1 and other allergens in the PR-10 protein family displayed by IgE targeting this epitope. Finally, we present the first high-resolution structure of a human allergen-specific IgE fragment in the single-chain fragment variable (scFv) format.

**Conclusions and Clinical Relevance:**

We here display the usefulness of allergen-specific human monoclonal IgE as a tool in studies of the crucial molecular interaction taking place at the initiation of an allergic response. Such studies may aid us in development of better diagnostic tools and guide us in the development of new therapeutic compounds.

## Introduction

A type I hypersensitivity response is initiated when allergen-specific IgE bound to high-affinity IgE receptors (FcεRI) on the surface of effector cells, such as mast cells and basophils, are cross-linked by allergen. This triggers a cascade of events eventually giving rise to the release of biologically active compounds that exert their effects both locally and systemically 1,2. It is thus evident that IgE plays a key role, as the allergen recognizer, in the mechanisms behind the symptoms associated with an allergic response, thereby influencing the quality of life for as many as one-third of the population of the industrialized world 3.

Over the years, a range of studies have characterized the genetic composition of human IgE repertoires, in terms of V(D)J germline gene usage, mutational status and evidence of antigenic selection 4–13. However, much work remains before we can understand the products of these antibody-encoding genes in terms of development and function.

One intriguing outcome of several of the past IgE-repertoire studies is a significant difference in the utilization of different immunoglobulin germline gene subgroups to produce the heavy chain variable (VH) domains of IgE in certain tissue as compared to IgE and other isotypes encoded by peripheral blood lymphocytes. More specifically, an overrepresentation of IgE-encoding transcripts derived from the immunoglobulin heavy variable (IGHV) 5 germline gene subgroup has been observed in such tissue samples 4–6. The reason behind and outcome of such biased use of certain genes are a matter of debate. Two possible explanations are that such skewed repertoires are the result of either a polyclonal expansion of B cells expressing surface IgE with origin in these genes, for example, by bacterial superantigens 14 or of a clonal selection process where certain allergens favour the selection of clones originating in a limited set of germline genes 10.

The second partner in the initial interaction triggering an allergic response, the allergen, adds further complexity to the task of understanding the molecular basis of the early events in such a response. Not only do most allergens exist as numerous different isoforms with varying potencies to provoke allergic reactions 15,16, but the existence of homologous allergens in related species also adds complexity to the analysis of development of human IgE repertoires. IgE-binding epitopes are to a certain extent shared between such homologous members of a protein family 17,18 explaining the often broad sensitivity profiles of allergic individuals. A detailed analysis of monoclonal human IgE has the potential to address these matters in great detail as we have carried out in the past for groups 1 and 5 grass pollen allergens 18.

The major birch pollen allergen, Bet v 1, is one of the most potent, and therefore also the most studied, tree allergens in Europe 19. It belongs to the widely represented PR-10 family [SCOPe database (http://scop.berkeley.edu/) entry d.129.3.1] 20 within the pathogenesis-related protein Bet v I family (Pfam database: http://pfam.sanger.ac.uk/family/PF00407). This allergen exists in at least 18 different isoallergens and isoforms, as defined by IUIS 21. Although several studies have dealt with characterization of responses between polyclonal IgE preparations and different Bet v 1 isoforms and homologues 15,16, the molecular basis of such interactions or discrimination of human IgE between PR-10 proteins of different origins still remains largely unresolved.

We here approach these issues and present a molecular characterization of Bet v 1-specific human antibody fragments derived from the IgE repertoire of allergic individuals. These antibody fragments have a genetic origin in IGHV5, the germline gene subgroup sometimes implicated as overrepresented in IgE repertoires 4–6. We show that such a repertoire of clonally different antibodies, despite their origin in a common immunoglobulin VH gene, is able to target more than one epitope on Bet v 1, thereby in principle fulfilling the prerequisites for cross-linkage and initiation of the allergic cascade. We also describe one of the epitopes recognized by such an antibody fragment with origin in the IgE repertoire in detail. From that assessment, we are able to define the molecular basis for the inability of this antibody fragment to recognize certain isoforms of Bet v 1 and to cross-react with many other members of the PR-10 subfamily.

## Methods

### Recombinant allergens and peptides

Recombinant Bet v 1.0101, Bet v 1.0102 (previously known as Bet v 1.0401), Mal d 1.0108, Aln g 1.0101 were obtained from Biomay (Vienna, Austria), while Bet v 1.0112 (previously known as Bet v 1.2801) and recombinant variants Bet v 1.2744 (Bet v 1.0112 carrying mutations N28T, L32Q, E45S and P108G) and Bet v 1.2595 (Bet v 1.0112 carrying mutations Y5V, E42S, E45S, N78K, K103V, K123I, K134E and D156H and an additional asparagine in position 160) 22 were received as a kind gift from ALK-Abelló (Hørsholm, Denmark). Bet v 1 residues are numbered in a way that excludes the N-terminal methionine, which is removed from the mature protein. Biotinylated peptides (BioTides) were purchased from JPT Peptide Technologies (Berlin, Germany). The 14-mer peptides used for the initial epitope mapping were designed to cover the Bet v 1.0101 sequence with an overlap of nine residues. Additional sequence-modified peptides were also synthesized. All peptides carried an additional glycine between the biotin and the N-terminal residue and an additional C-terminal glycine residue.

### Isolation of Bet v 1-specific antibody fragments

A combinatorial single-chain fragment variable (scFv) library, based on transcripts encoding the VH domain of IgE origin derived from nasal tissue of two allergic donors 4, was constructed essentially as described by Andréasson et al. 8. Phage display selections from the scFv library were performed on Bet v 1 isoforms Bet v 1.0101, Bet v 1.0102 and Bet v 1.0112. After three rounds of selections in Immunotubes (Nunc, Roskilde, Denmark) coated with allergen diluted to 5 μg/mL in PBS, single colonies were randomly chosen for further analysis.

### Construction of vectors for production of scFv and scFv-CHε2-4 fusion proteins

To allow for the production of soluble scFv, not displayed on phage, genes encoding isolated scFv were transferred in a single cloning step into a production vector 23. After ligation, the DNA was transformed into chemically competent One Shot Top10 *Escherichia coli* (Invitrogen, Carlsbad, CA, USA).

To enable the production of scFv-Fcε fusion proteins, an antibody format previously employed and evaluated in other studies 18,24, the scFv-encoding genes were cloned, using *Nco*I and *Not*I, into a vector carrying a sequence encoding the second, third and fourth constant domains of human IgE 18. The ligated DNA was transformed into chemically competent XL1-Blue *E. coli* (Agilent Technologies, Santa Clara, CA, USA).

### Production of scFv in E. coli

After transfection, cells carrying the vector encoding scFv were grown in 2xYT-media (supplemented with 100 μg/mL carbenicillin) until OD_600_ = 0.9 was reached. At this point, protein production was induced by addition of isopropyl β-D-1-thiogalactopyranoside to a final concentration of 1 mm. After 16 h of production at 30°C, the cells were harvested by centrifugation at 7500 × g for 12 min and treated with lysozyme (Sigma-Aldrich, St. Louis, MO, USA). Soluble proteins were purified using affinity chromatography with Ni-NTA agarose columns (Qiagen, Hilden, Germany).

### Production of scFv-Fcε fusion proteins in HEK293 cells

HEK293 cells were grown in minimum essential medium (Invitrogen) supplemented with 2 mm L-glutamine and 10% HyClone fetal bovine serum (Hyclone Laboratories, South Logan, UT, USA) in 5% CO_2_ at 37°C. At 90% confluency, the cells were transfected with vectors encoding the scFv-Fcε fusion proteins using Lipofectamine 2000 (Invitrogen). Supernatants containing scFv-Fcε fusion proteins were collected after 72 h and sterilized by filtration (0.45 μm) prior to analysis.

### ELISA binding assays

The ability of the isolated Bet v 1-specific IgE-derived clones, either displayed on phage, as soluble scFv or as scFv-Fcε fusion proteins to bind to recombinant allergens was determined using ELISA. Allergens and BSA (negative control) were diluted to 5 μg/mL in PBS and coated in microtiterplates (Corning). Blocking was performed with 1% w/v BSA, 0.05% Tween-20 in PBS. Bound phages were detected with horseradish peroxidase (HRP)-conjugated anti-M13 mAb (GE Healthcare, Piscataway, NJ, USA), scFv with an HRP-labelled anti-FLAG M2 mAb (Sigma-Aldrich) and scFv-Fcε fusion proteins with an HRP-labelled anti-IgE antiserum (KPL, Guildford, UK) using 1-Step Ultra TMB – ELISA Substrate (Pierce, Rockford, IL, USA) as chromogen. Absorbance was measured at 450 nm.

The analysis of reactivity of scFv-Fcε fusion proteins to biotinylated peptides was performed essentially in the same manner, with the exception that the microtiterplates were precoated with 1 μg/mL streptavidin in PBS prior to the addition of the biotinylated peptides diluted to 1 μg/mL in PBS. After 1 h of incubation at 37°C, unbound peptides were washed away and diluted supernatants containing scFv-Fcε fusion proteins were added. Detection was performed as described above.

### Biacore blocking assay

Recombinant Bet v 1.0101 was coupled (2200 RU) to a CM5 sensor chip (GE Healthcare) using a standard amine coupling protocol. Samples were diluted in HBS-EP running buffer (10 mm HEPES pH 7.4, 150 nm NaCl, 3 mm EDTA, 0.005% v/v surfactant P20). Blocking assays were performed by injection of the blocking scFv (200 μg/mL) for 300 s followed by injection of the secondary scFv (50 μg/mL) for 120 s. As a reference, HBS-EP running buffer was injected and followed by the secondary scFv as above.

### Genetic analysis

Sequencing of genes encoding Bet v 1-specific IgE was performed by GATC Biotech (Konstanz, Germany). The genetic origin of sequences encoding the heavy and light chain variable (VL) domains, and mutational status of the transcripts was determined using the IMGT/V-QUEST web tool (program version 3.2.20; reference directory release: 201135-3) 25. Genes were annotated according to the IMGT nomenclature 26, which is used throughout this manuscript. Sequence alignments were performed using MacVector 12.0.3 (MacVector, Cory, NC, USA).

### Crystallization of the M0418 scFv

Crystals were grown using the sitting drop vapour diffusion method in MRC 96-well plates. The reservoir comprised 50 μL of 0.1 m MES pH 6.5 and 25% (w/v) PEG 4000. The drops contained 200 nL of protein, at a concentration of 1.4 mg/mL, and 70 nL of reservoir, and were kept at 18°C. Crystals typically started to appear after a few days and were cryoprotected in 0.1 m HEPES pH 7.5, 25% (w/v) PEG 4000 and 18% (w/v) ethylene glycol before flash cooling in liquid nitrogen.

### Data collection, structure determination and refinement

Data were collected at beamline I02 at the Diamond Light Source (Harwell, UK). Data were integrated with XDS 27 implemented in the *xia2* package 28 and further processed with the CCP4 suite of programs 29. The structure was solved with MOLREP 30 using protein atoms from Protein Data Bank (PDB) entry 2YC1 31 as a search model, and two molecules were located in the asymmetric unit. Refinement was performed with PHENIX 32 and alternated with rounds of manual model building with *Coot* 33. The final model comprised protein residues 1-127 and 143-252 from chain A, protein residues 1-101, 108-126 and 143-254 from chain B, four ethylene glycol molecules, one polyethylene glycol molecule, and 409 water molecules. Neither the linker region between the H and L chains nor the C-terminal region of the scFv containing the Flag-tag and His-tag were built. Electron density at the N-terminus of both protein chains suggested the conversion of N-terminal glutamine to pyroglutamate. The quality of the model was assessed with MolProbity 34 and POLYGON 35, both implemented in PHENIX. Data processing and refinement statistics are reported in Table1. Co-ordinates and structure factors have been deposited at the PDB with codes 4buh and 4buhsf, respectively. The coordinate file for the M0418 scFv construct, incorporating the VH domain, (Gly_4_Ser)_3_ linker, VL domain, Flag-tag and His-tag, is numbered sequentially.

**Table 1:** Data processing and refinement statistics

Data processing	
Space group	*C* 1 2 1
Unit cell dimensions (Å)	a = 99.50, b = 76.42, c = 70.18, β = 118.07°
Resolution*	27.30–1.30 (1.37–1.30)†
Completeness*	96.1 (93.7)†
Redundancy*	9.1 (7.8)†
Mean ((*I*)/σ(*I*))*	12.2 (3.5)†
R_merge_ (%)*	11.1 (87.4)†
Refinement	
R_work_ / R_free_ (%)‡	14.53 / 17.28
No. of reflections	109 029
RMSD	
Bond lengths (Å)	0.006
Bond angles (°)	1.169
Coordinate error (Å)	0.11
No. of atoms	
Protein	3694
Solvent	409
Other§	34
Ave. *B* factor (Å^2^)	
Protein	16.33
Solvent	31.09
Other§	26.85
Ramachandran plot	
Favoured (%)	97.8
Allowed (%)	100

* Data scaled with SCALA 36 from the CCP4 suite 29.

† Numbers in parentheses are for the highest resolution shell.

‡ R_free_ set comprising 5% of reflections.

§ Four ethylene glycol molecules and one polyethylene glycol molecule.

## Results

### Isolation of Bet v 1-specific antibody fragments from the IgE repertoire

**Figure 1:**
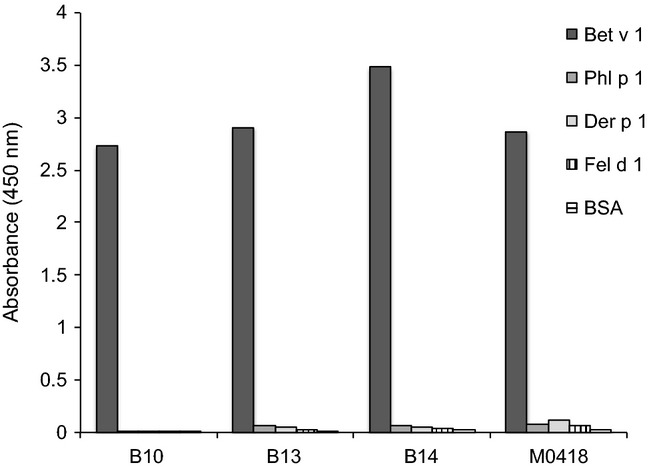
Binding specificity of novel scFv selected on Bet v 1 as determined by phage-ELISA on a panel of different allergens and BSA. All scFv are highly Bet v 1-specific and show no detectable cross-reactivity. All samples were run in duplicate.

To enable characterization of molecular interactions between antibody fragments with origin in the human IgE repertoire and the major birch pollen Bet v 1, we performed phage display selections on the three Bet v 1 isoforms Bet v 1.0101, Bet v 1.0102 and Bet v 1.0112 from an antibody fragment library created from atopic donors. The library was created from transcripts encoding the VH domain of IgE isolated from nasal tissue of two allergic subjects known to have an IgE repertoire overrepresented by antibodies derived from the IGHV5 gene subgroup 4. These selections generated four novel-specific binders (B10, B13, B14 and M0418). Three binders, B10, B13 and B14, were isolated by selection on the major isoform Bet v 1.0101, while selection on isoform Bet v 1.0102 generated one additional binder (M0418) together with re-isolation of binder B14. Selections performed on isoform Bet v 1.0112 failed to generate any binders. All isolated scFv were highly Bet v 1-specific, showing no cross-reactivity to a panel of unrelated allergens (Fig.1).

### Selected Bet v 1-specific antibody VH-encoding sequences are derived from the IGHV5 germline subgroup

**Figure 2:**
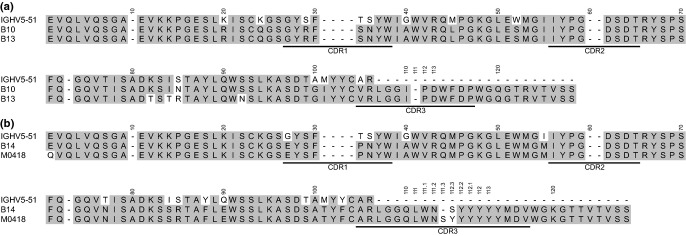
Sequence alignment of the VH of the four Bet v 1-specific IgE (a; B10 and B13, b; B14 and M0418) together with a translation of the IGHV5-51 germline gene, from which all clones originate. CDR1-3 are underlined.

Genetic analysis of VH-encoding sequences of the four Bet v 1-specific antibody fragments reveals that they all originate in the IGHV5 germline subgroup (Table2), a subgroup previously shown to be overrepresented in some IgE-encoding transcriptomes of allergic individuals 4–6. They all originate in the relatively common IGHV5-51 H chain V gene 37, but vary in terms of length of the rearrangement encoding the third complementarity determining region of the H chain (CDRH3) (12-19 codons). Clones B10 and B13 originate from the same VH chain rearrangement and share the same CDRH3, considered to be of crucial importance for antibody specificity 38. They also share some but not all of their substitutions (Fig.2a and Figure S1), suggesting that they have diversified from common precursor through a mutational process. Likewise, clones B14 and M0418 have very similar heavy chain sequences (Fig.2b), primarily differing only by the addition of one codon in the CDRH3 of M0418. They are thus also likely to share a common ancestor. The light chain sequences of these scFv are all diverse (Table2, Figure S2), but they all belong to the λ light chain class of variable domains.

**Table 2:** Genetic origin of Bet v 1-specific IgE

		H chain germline gene origin				L chain germline gene origin		
ScFv clone	GenBank accession numbers	IGHV	IGHD	IGHJ	CDRH3 length	IGLV	IGLJ	CDRL3 length
B10	KF240714	IGHV5-51	IGHD3-16	IGHJ5	12	IGLV2-14	IGLJ1	10
B13	KF240715	IGHV5-51	IGHD3-16	IGHJ5	12	IGLV2-23	IGLJ3	11
B14	KF240716	IGHV5-51	IGHD5-5	IGHJ6	18	IGLV3-1	IGLJ1	10
M0418	KF240717	IGHV5-51	IGHD5-5	IGHJ6	19	IGLV1-44 or IGLV1-47	IGLJ3	11

### Isoform reactivity of Bet v 1-specific human scFv from the IgE repertoire

**Figure 3:**
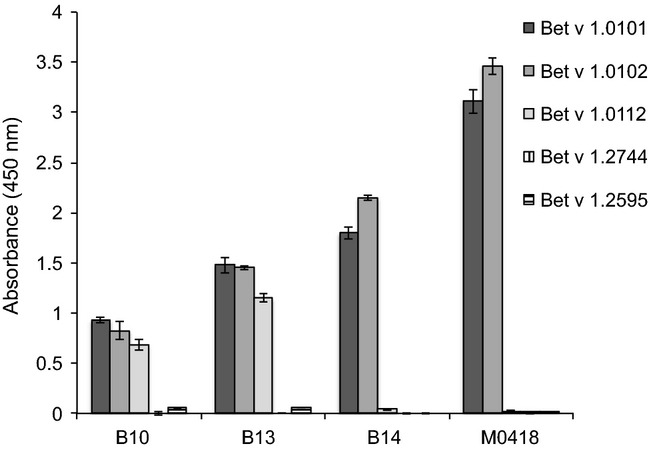
Reactivity profiling of the four scFv against the three naturally occurring isoforms Bet v 1.0101, Bet v 1.0102 and Bet v 1.0112 and the artificial isoforms Bet v 1.2744 (Bet v 1.0112 carrying mutations N28T, L32Q, E45S and P108G) and Bet v 1.2595 (Bet v 1.0112 carrying mutations Y5V, E42S, E45S, N78K, K103V, K123I, K134E and D156H and an additional asparagine at position 160) 22. All samples were run in duplicate and error bars represent one SD.

Different Bet v 1-isoforms have shown varying ability to initiate allergic responses 15,16 and not all IgE-binding epitopes may be shared between the different isoforms, a fact that potentially could complicate diagnosis and specific immunotherapy. We therefore profiled the cross-reactivity between our human Bet v 1-specific IgE-derived scFv and three naturally existing Bet v 1-isoforms (Fig.3). All four scFv, including M0418 that was selected on Bet v 1.0102, did bind the major isoform Bet v 1.0101. It was thus not possible to select binders from this combinatorial IgE library without cross-reactivity to Bet v 1.0101, indicating that epitopes carried on this isoform were major contributors in the sensitization of the allergic donors to Bet v 1-like allergens. Notably, all four scFv showed high reactivity with Bet v 1.0102, an isoform suggested to be a naturally occurring hypoallergen (i.e. a naturally existing Bet v 1-isoform with reduced allergenicity) 15,16,39. In contrast, only two of the available Bet v 1-specific scFv, B10 and B13, showed binding to Bet v 1.0112, another naturally existing variant only differing from Bet v 1.0101 by a single amino acid substitution (F62L). Bet v 1.0112 has also been the foundation for design of two hypoallergenic variants, Bet v 1.2595 and Bet v 1.2744 22, carrying 4 and 9 mutations, respectively, to which none of our four scFv showed any detectable binding, supporting their hypoallergenic properties.

### Bet v 1-specific scFv bind two non-overlapping epitopes

As the allergic response is initiated by cross-linkage of high-affinity IgE receptors (FcεRI) on effector cells, such as mast cells or basophils, IgE targeting of at least two non-overlapping epitopes is required, unless the allergen is oligomeric. We therefore investigated, using Biacore blocking assays, whether or not the isolated clones fulfilled this criterion. Indeed, clones B14 and B13 (as scFv) were each shown to target two distinctly separated epitopes in this assay (Figure S3). Bearing in mind the sequence similarities between clones B14 and M0418, and between clones B10 and B13, it is likely that members of each pair target the same, or very similar, epitopes. It thus seems as if these four IgE-derived clones, all with origin in IGHV5, do recognize two independent epitopes on the allergen and consequently meet one criterion needed for initiation of an allergic cascade.

### Epitope mapping of Bet v 1-specific antibody fragments with peptides

**Figure 4:**
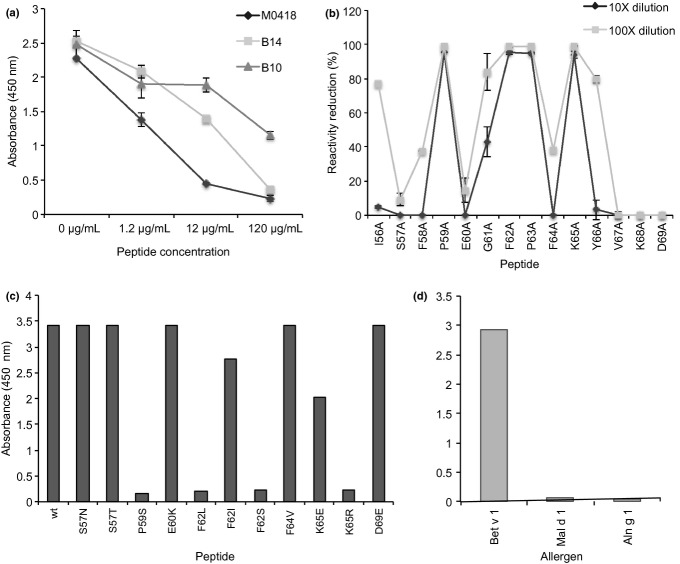
Peptide mapping of epitopes recognized by Bet v 1-specific scFv-Fcε fusion proteins and reactivity profiling of IgE clone M0418. A peptide encompassing residues I56 to D69 of Bet v 1.0101, recognized by the two sequence-related IgE clones M0418 and B14, and an additional peptide spanning residues G26 to S39, recognized by B10, had been identified (Figure S4). Inhibition of binding of these scFv-Fcε to immobilized Bet v 1 by respective peptides confirmed the reactivity of these scFv-Fcε to these parts of the allergen (a). Alanine-scanning modifications of residues within this sequence defines the importance of each residue for reactivity with M0418 (b). Further, reactivity profile of M0418 against the identified Bet v 1.0101 peptide I56 to D69 and modified versions thereof was performed (c). Modifications include some of those found in isoforms of Bet v 1, in homologues of Bet v 1 found in other Betula species, or in PR-10 family proteins of other plant species. Critical amino acid differences that explain an inability of M0418 to bind different isoforms/homologues are apparent, as illustrated by the lack of reactivity (d) against Mal d 1.0108 and Aln g 1.0101 (both of which among other differences carry the F62S mutation). Samples were run in duplicate and error bars represent one standard deviation.

To map the epitopes of the available scFv-CHε2-4 fusion proteins, their ability to bind to a set of peptides covering the amino acid sequence of Bet v 1.0101 was assessed by ELISA (Figure S4). Clone M0418 showed strong and B14 showed weak reactivity to a peptide covering residues I56 to D69, while B13 bound weakly to a peptide covering residues G26 to S39. B10 failed to bind any of the tested peptides, but, as mentioned above, should likely bind the same epitope as B13. These binding specificities were confirmed by testing the ability of the identified peptides in soluble form to inhibit binding to immobilized Bet v 1 (Fig.4a).

The epitope of M0418 was further defined by ELISA. The individual importance of residues within the I56-D69 for the interaction was evaluated using alanine-scanned versions of the peptide (Fig.4b). The M0418 construct demonstrated a reactivity profile in which residues P59, F62, P63 and K65 were most critical for the interaction; I56, G61 and Y66 were of intermediate importance while several others, for example, S57 and E60, were less important. In agreement with these findings, further studies involving peptide sequences encompassing shorter segments of the Bet v 1-derived peptide defined the sequence I56-K65 to be most important for the interaction (Figure S4). These residues reside in the turn connecting β-strands 3 and 4 of Bet v 1, a turn that is at a distance from residues important for recognition of Bet v 1 by the BV16 mouse monoclonal IgG and another previously defined human IgE scFv (Figure S5) 40,41.

Additional peptide variants carrying modifications typical of isoforms of Bet v 1 and related proteins found in other plant species were also investigated. Notably, an S57N modification, that differentiates Bet v 1.0101 from Bet v 1.0102 in this part of the sequence, was compatible with peptide binding by M0418 (Fig.4c), in agreement with the finding that M0418 cross-reacts to Bet v 1.0102. Further analysis illustrated that modification of F62, one of the most diverse residues among the various isoforms of Bet v 1 (Figure S6), into serine and leucine was not compatible with binding to M0418 while a peptide carrying a F62I modification retained some ability to bind M0418. Notably, the critical importance of residue F62 in this interaction explains the inability of M0418 to bind Bet v 1.0112, an isoform that carries a single modification (F62L) in relation to Bet v 1.0101. Altogether, M0418 and other antibodies that detect this epitope in a similar way should be expected to recognize most but not all of the isoforms of the isoallergens Bet v 1.01 (i.e. those that carry F62 and potentially those that carry I62) but none of the members of the Bet v 1.02 isoallergen group. (Figure S6).

Bet v 1 analogues found in other plant species may cross-react with antibodies specific for the major birch allergen. Such allergens derived from a range of plants differ in the part of the sequence important for binding of M0418 to Bet v 1 (Figure S7). In agreement with this, M0418 did not recognize Mal d 1.0108 or Aln g 1.0101, as determined by ELISA (Fig.4d). M0418 is thus a member of the Bet v 1-specific immune response that shows limited cross-reactivity to other allergens of the PR-10 family.

### Structure of M0418 scFv specific for Bet v 1

**Figure 5:**
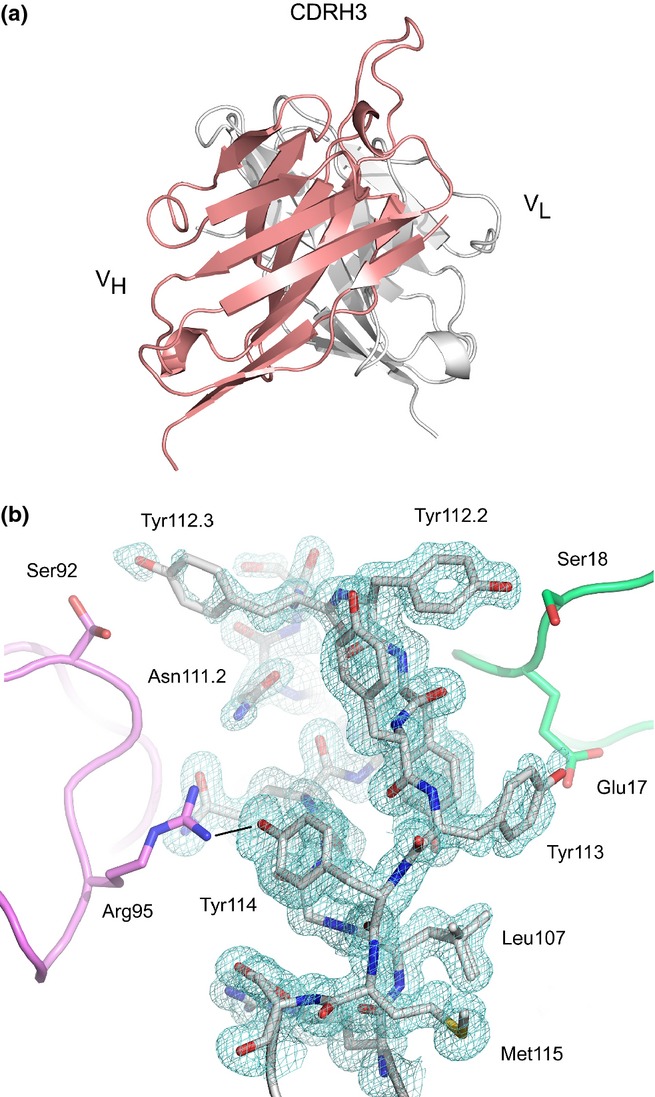
(a) Overall structure of the M0418 scFv. The disposition of CDRH3 above the surface of molecule A is shown, and the H and L chain V domains are coloured in salmon and white, respectively. (b) CDRH3 from molecule A. A 2F_o_-F_c_ electron density map is contoured at 1.0σ around residues from CDRH3. Two symmetry-related copies of molecule B (pink and green) flank CDRH3 on either side. Residues from CDRH3 molecule A are shown as a stick representation, as are selected residues from the two symmetry-related molecules, and the two alternative conformations modelled for Leu107 (VH) and Ser92 (VL) are also shown. A hydrogen bond between Tyr114 (VH) Arg95 (VL) is indicated with a black line. The figure was produced with PyMOL 42.

The crystal structure of the M0418 scFv was determined to 1.3Å resolution. The structures of the two independent molecules of the asymmetric unit were similar and were superposed with an RMSD value of 0.56Å over 232 Cα atoms. The most significant difference between the two molecules was disorder of a stretch of residues from CDRH3 of molecule B (residues 110-112.3), while that from molecule A was completely modelled. In molecule A, CDRH3 extends ˜8Å above the surface of the scFv (Fig.5a).

The order/disorder observed for CDRH3 in the two molecules can be attributed to differences in crystal packing. In molecule A, CDRH3 is flanked on either side by two symmetry-related molecules (Fig.5b). On the one side, the first symmetry-related molecule (coloured green in Fig.5b) forms hydrogen bonds (including water-mediated hydrogen bonds) and van der Waals interactions with residues Leu111, Tyr112.2, Tyr112.1 and Tyr113 from CDRH3. On the other side, the second symmetry-related molecule (coloured in pink in Fig.5b) forms hydrogen bonds (including hydrogen bonds mediated by an ethylene glycol molecule) and van der Waals interactions with residues Gln110, Trp111.1, Asn111.2, Tyr112.3 and Tyr114.

However, the environment of CDRH3 from molecule B is different. A symmetry-related molecule (in an equivalent position to that forming the first interaction described for molecule A) preserves the interaction with Tyr112.1 and Tyr113 on one side, although the side chain for Tyr108 is disordered, and the position of backbone atoms for residues 112.2 and 112.1 is shifted by ˜2Å. On the other side, CDRH3 is not flanked by a symmetry-related molecule, and residues 110-112.3 are disordered.

Thus, in the absence of stabilizing contacts, CDRH3 is mobile. Two glycine residues immediately precede the CDRH3 loop, at positions 108 and 109, and presumably contribute to its flexibility.

## Discussion

Despite the proven usefulness of human monoclonal antibodies in studies of biomedically important antibody responses, for instance in viral and bacterial infection as well as in autoimmunity, researchers within the field of allergic disease have only recently started to make use of the huge potential contained in human monoclonal IgE. We have in two recent studies deconvoluted the information content of two sets of human monoclonal IgE-derived scFv specific for the two major grass pollen allergens Phl p 1 18 and Phl p 5, respectively, allowing for in-depth studies of the interaction between such antibody fragments and their corresponding allergens. On the same path, others have utilized both human and murine monoclonal antibodies aiming at a deeper understanding of the molecular interactions taking place at the initiation of an allergic response (exemplified by 43–47). Studies of these kinds have, among other things, led to identification of new IgE-binding epitopes and a better understanding of aspects of the IgE response that define the biological effects exerted by such antibodies. We in this study continue on that path by presenting isolation of four novel human antibody fragments with origin in the IgE repertoire, all specific for the major birch allergen Bet v 1, and characterization of their interaction with the allergen.

Since the structure of Bet v 1 was solved almost two decades ago 48, numerous studies have identified single amino acids, peptide stretches or surface patches that to some extent are involved in the interaction between human IgE and the allergen. Recently, Hecker et al. 41 reported the first human IgE-binding epitope on Bet v 1 recognized by a scFv based on a VH with human IgE origin and a synthetic VL. However, the two IgE epitopes we report within this study are both distinctly different from the epitope reported by Hecker et al., and unlike previously characterized IgE antibodies several of the binders included in this study target epitopes that to some extent can be defined by short linear peptides. The more narrowly defined epitope of IgE-derived clones M0418 and B14 (residues I56 to K65) encompasses the loop connecting β-strands 3 and 4 of Bet v 1, and belongs to a conformationally flexible region of the allergen 49. This epitope partially overlaps that of the mouse monoclonal IgG1 antibody mAb2 shown to exhibit a substantial inhibition of binding of human serum IgE to Bet v 1 45. In addition, earlier studies using mouse monoclonal antibodies derived by immunization with birch pollen identified this area as important for IgE binding 43. The second novel epitope described in this study, covering part of α-helix 2 and the loop connecting it to β-strand 2, belongs to a more structurally conserved region of the allergen and is recognized by B10 and most likely B13. This epitope resides in an area previously not identified as important for human IgE binding using human or murine monoclonals, but in vitro mutagenesis studies did identify the phenylalanine in position 30 to have influence on IgE binding 50. It is, however, difficult to conclude whether this mutation affects IgE binding directly or via induction of conformational changes in the protein.

The identification of novel and more precise characterization of such biologically relevant epitopes made possible by usage of antibody fragments, derived from human IgE repertoires, provides useful information that could aid in rational design of new hypoallergenic versions of Bet v 1 to be used as safer alternatives to the allergen extracts currently used in specific immunotherapy. The epitopes identified by us and previously by Hecker and colleagues 41 should be suitable targets for directed mutagenesis in attempts to reduce the IgE reactivity of the protein, in a strategy previously described for the major timothy grass pollen allergen Phl p 1 18. Further, detailed knowledge on the location of IgE-binding epitopes would be valuable in future attempts to increase the resolution of allergy diagnosis, from component-resolved to epitope-resolved diagnosis.

Although adjacent to one another on the surface of Bet v 1, the two identified epitopes could allow for simultaneous binding of two scFv targeting their respective epitopes (Figure S3), thereby fulfilling the basic criteria of FcεRI cross-linkage. The optimal separation distance for such FcεRI cross-linkage, as determined by degranulation assays, is suggested to be 44-51Å 51, which may be rationalized by the disposition of Fabs in the asymmetrically bent FcεRI bound form of IgE 52. In the Bet v 1 monomer, the most distant Cα atoms from the M0418/ B14 and B13 epitopes are only ˜20Å apart. However, the ability of Bet v 1 to cross-link receptor on effector cells was shown to require a dimer rather than a monomer 53. In the model of the proposed Bet v 1 dimer (Figure S8) 54, the distance between Cα atoms of Phe62, a critical residue for M0418 epitope, is 40Å, approaching the favourable distance for FcεRI cross-linking 52. While the distance between the M0418 and B13 epitopes of different Bet v 1 molecules within the dimer varies from ˜13-35Å, conformational flexibility of the M0418 epitope, together with the protruding CDRH3 of the antigen binding site of M0418 (Fig.5), could aid in optimal positioning of receptors on the cell surface, when different epitopes are engaged in cross-linking.

The presence of skewed IgE repertoires, with overrepresentation of VH-encoding transcripts with origin in only certain germline genes or gene subgroups, has been suggested in several previous studies 4–6, but we here, to our knowledge for the first time, show that such a repertoire, here restricted to the usage of transcripts with origin in IGHV5-51, despite its theoretical loss of diversity of antigen binding sites, still should be able to initiate allergic responses. Although there is a positive correlation between the clonal size of allergen-specific IgE and the extent of the biological effect (i.e. basophil degranulation), a single pair of IgE antibodies, targeting non-overlapping epitopes 55, as is the case with M0418/B14 and B10/B13, is enough to achieve a strong degranulation of effector cells.

The four IgE-derived antibody fragments included in this study were, as all other allergen-specific human monoclonal IgE available to date, selected from combinatorial antibody libraries 56. The usage of such antibody fragments has been successfully employed in several previous studies of human IgE 9,10,17,18,40,57 and of responses involving other human isotypes and relies on the fact that the random pairing of sequences encoding the VH domain of human IgE with random sequences encoding VL domain can recreate VH-VL pairs with antigen specificity. As a large portion of the specificity of antibodies resides within the VH 58, especially in the CDRH3 38, it should be considered likely that naturally occurring specificities are largely retained among selected antigen-specific binders. One should, nevertheless, be aware of the potential risk of creating in particular slightly modified specificities that do not match those found *in vivo* through the random pairing of VH and VL. The fact that several similar scFv with quite different VL were isolated in this study argues in favour of the presence of specificity determining VH domains that have originally been selected *in vivo*.

Access to human monoclonal IgE allows for extensive characterization of such antibodies at a much more detailed resolution than that afforded by studies of polyclonal serum IgE preparations. As human and mouse antibody responses, and human IgE and IgG responses have been shown to differ in many respects 18,59, availability of human monoclonal IgE offers new opportunities to more faithfully study antibody–allergen interactions critical to allergic disease. Only in a few instances 46,47 has it been possible to study human IgE–allergen interactions using X-ray crystallography. In this study, we were able to obtain a first high-resolution structure of a human allergen-specific IgE fragment in the scFv format, a format that may be more readily used in the selection of specific binders as compared to Fab fragments used in the past to determine human IgE–allergen interactions. Unfortunately, despite efforts using both intact allergen and allergen-derived peptides, it was not possible to obtain crystals of a complex, attributed to the involvement of CDRH3 in maintaining crystal contacts. Nevertheless, the structure of the unbound IgE scFv M0418 demonstrates that a human allergen-specific antigen binding site may display a protruding CDRH3, similar to human antibodies of other isotypes 60–62.

Even in the absence of allergen-IgE scFv structures, the availability of human IgE-derived antibody fragments allows for very detailed analyses of human IgE–allergen interactions at a level not easily obtained with polyclonal antibodies. In this study, we have defined two human IgE epitopes on Bet v 1 and we have been able to define their very different cross-reactive potential in terms of recognition of Bet v 1 isoforms and other proteins of the PR-10 family. Importantly, it has also been possible to connect genetic studies of IgE-repertoire composition and its involvement in responses against an important allergen family. We foresee that human monoclonal IgE as they become more readily available will continue to prove their extensive value in defining allergy-causing immune responses and in providing new solutions (e.g. new hypoallergenic variants as described in the past 18) that may be used to treat allergic disease in the clinic.

In summary, we have in this study isolated four novel Bet v 1-specific antibody fragment with origin in human IgE repertoires and defined two peptides, each containing the epitope of one of the two clonally related IgE pairs. We have identified certain allergen residues that are crucial for the interaction with one of these human IgE-derived antibody fragments, providing an explanation, at a molecular level, for the inability of this allergen-specific IgE fragment to recognize certain allergen isoforms and to cross-react with many members of the PR-10 protein family. We also show that IgE repertoires restricted in their genetic composition, in this case to the IGHV5-51 germline gene, a major member of a gene subgroup reported 4–6 to be overrepresented in some IgE responses, still retains the ability to generate IgE capable of receptor cross-linkage. We foresee that the strategy used in this and other studies making use of human monoclonal allergen-specific IgE will help us decipher unanswered questions concerning the crucial interactions between IgE and the allergen at the onset of allergic reactions.

## Supporting Information

Figure S1 Sequence alignment of the VH of the four Bet v 1 specific IgE (A; B10 and B13, B; B14 and M0418) together with the V, D and J germline gene segments, from which the clones have their origin. CDR1-3 are underlined **Figure S2.** Sequence alignment of the VL of the four Bet v 1-specific IgE B10, B13, B14 and M0418. CDR1-3 are underlined **Figure S3.** Biacore inhibition assays of scFv clones B13 (A) and B14 (B) to demonstrate differences in epitope recognition **Figure S4.** Epitope mapping of human monoclonal IgE M0418 (A), B13 (B) and B14 (C) (as scFv-Fcε fusion proteins) by ELISA against peptides covering the amino acid sequence of Bet v 1.0101 **Figure S5.** Mapping of epitopes on Bet v 1 defines three of the regions recognized by antibodies **Figure S6.** Sequence (A) of 18 isoallergens/isoforms of Bet v 1 and variability (B) in the different residues calculated as described by Wu and Kabat [S4] **Figure S7.** The sequence recognized by M0418 (Bet v 1 residues I56 to D69) exemplified in some members of the PR-10 family of proteins. Identical residues are shaded and indicated by a dot **Figure S8.** Proposed model of dimeric Bet v 1 [S5]. The epitope targeted by clone M0418 (residues 56-66) is coloured in pink while that targeted by B13 (residues 26-39) is coloured in blue
