# Supplementary material for: Human IgE against the major allergen Bet v 1 – defining an epitope with limited cross-reactivity between different PR-10 family proteins
Source: Clin Exp Allergy. 2014 Jan 22;44(2):288–99. doi: 10.1111/cea.12230 (PMC4215112; doi:10.1111/cea.12230)
Supplement: Figure S1 — Sequence alignment of the VH of the four Bet v 1 specific IgE (A; B10 and B13, B; B14 and M0418) together with the V, D and J germline gene segments, from which the clones have their origin. CDR1-3 are underlined Figure S2. Sequence alignment of the VL of the four Bet v 1-specific IgE B10, B13, B14 and M0418. CDR1-3 are underlined Figure S3. Biacore inhibition assays of scFv clones B13 (A) and B14 (B) to demonstrate differences in epitope recognition Figure S4. Epitope mapping of human monoclonal IgE M0418 (A), B13 (B) and B14 (C) (as scFv-Fcε fusion proteins) by ELISA against peptides covering the amino acid sequence of Bet v 1.0101 Figure S5. Mapping of epitopes on Bet v 1 defines three of the regions recognized by antibodies Figure S6. Sequence (A) of 18 isoallergens/isoforms of Bet v 1 and variability (B) in the different residues calculated as described by Wu and Kabat [S4] Figure S7. The sequence recognized by M0418 (Bet v 1 residues I56 to D69) exemplified in some members of the PR-10 family of proteins. Identical residues are shaded and indicated by a dot Figure S8. Proposed model of dimeric Bet v 1 [S5]. The epitope targeted by clone M0418 (residues 56-66) is coloured in pink while that targeted by B13 (residues 26-39) is coloured in blue [file cea0044-0288-sd1.pdf]

Supporting Information

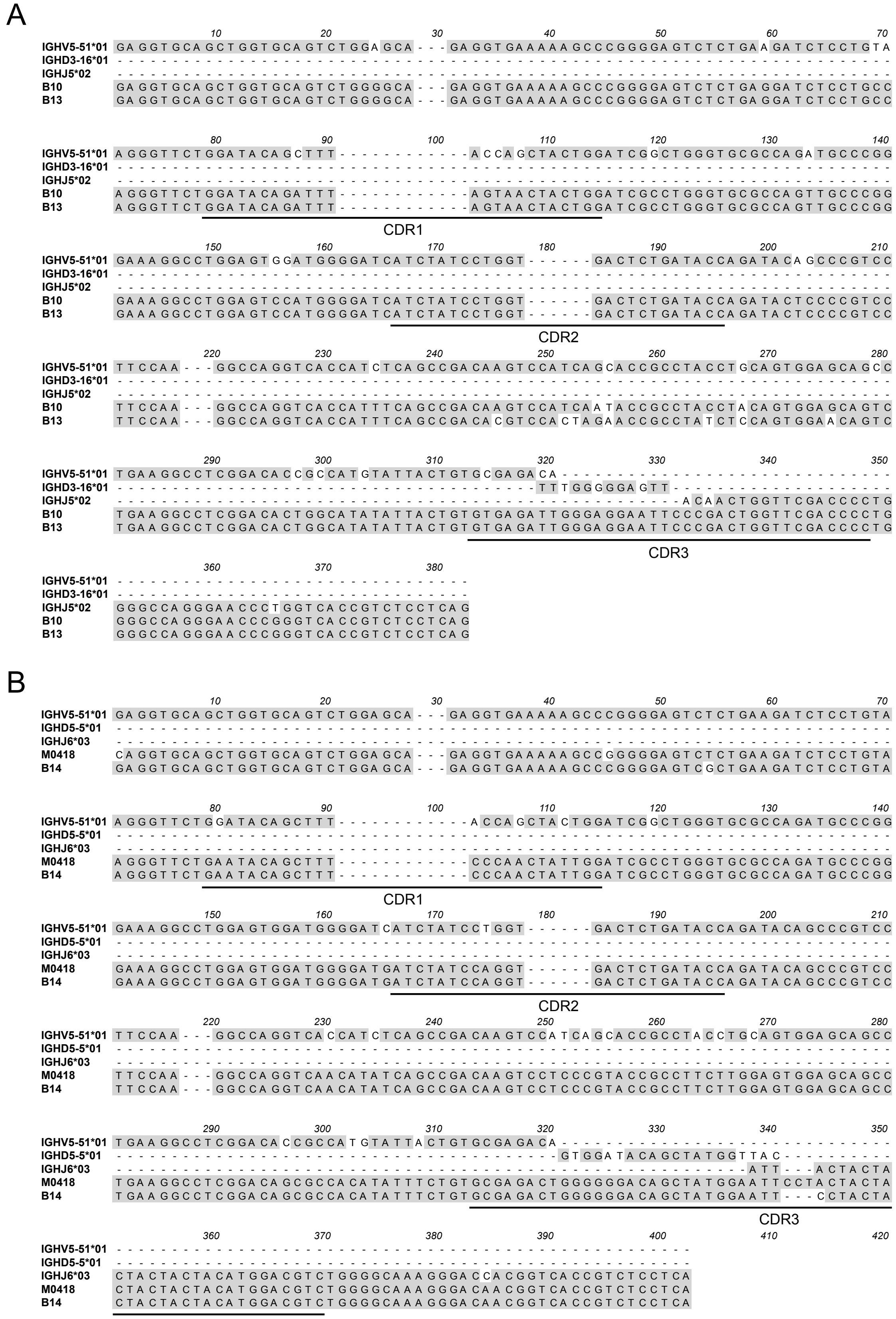

Figure S1. Sequence alignment of the VH of the four Bet v 1 specific IgE (A; B10 and B13, B; B14 and M0418) together with the V, D and J germline gene segments, from which the clones have their origin. CDR1-3 are underlined.

```
                        10                  20                  30                  40                  50
B10     Q S V L T Q P A S - V S G S P G Q S I T I S C T G T S S D V G - - - D Y N Y V S W Y Q Q H P G K A P
B13     S Y E L T Q P P S - V S G S P G Q S V T I S C T G T S S D V G - - - G Y N L V S W Y Q Q H P G K A P
B14     Q S V L T Q P P S - V S V S P G Q T A R I T C S G D K L G - - - - - - N K Y A A W Y Q Q K P G Q S P
M0148   S Y E L T Q P P S - A S G T P G Q R V T I S C S G S S S N I - - - - G G N T V N W Y Q Q V P G T A P
                                                        CDR1

                        60                  70                  80                  90                 100
B10     K L L I Y Q V - - - - - - - S N R P S G V S - S R F S G S K - - S G N T A S L T I S G L Q A E D E A
B13     R L M I Y E D - - - - - - - T K R P S G V S - H R F S G S K - - S G N T A S L T I S G L Q A E D E A
B14     V L V I Y Q D - - - - - - - S K R P S G I P - E R F S G S N - - S G N T A T L T I S G T Q A M D E A
M0148   R L L I Y K N - - - - - - - N Q R P S G V P - D R F S G S K - - S G T S A S L A I S G L R S E D E A
                  CDR2

                       110                 120
B10     D Y Y C T S Y T S - - - S S T Y V F G T G T K L T V L G
B13     D Y Y C Y S Y A T G - - R P S R L F G G G T K L T V L G
B14     D Y Y C Q A W D S - - - S T F Y V F G T G T K L T V L G
M0148   D Y Y C E A W D G G - - L R G G V F G G G T K L T V L G
                          CDR3
```

Figure S2. Sequence alignment of the VL of the four Bet v 1-specific IgE B10, B13, B14 and M0418. CDR1-3 are underlined.

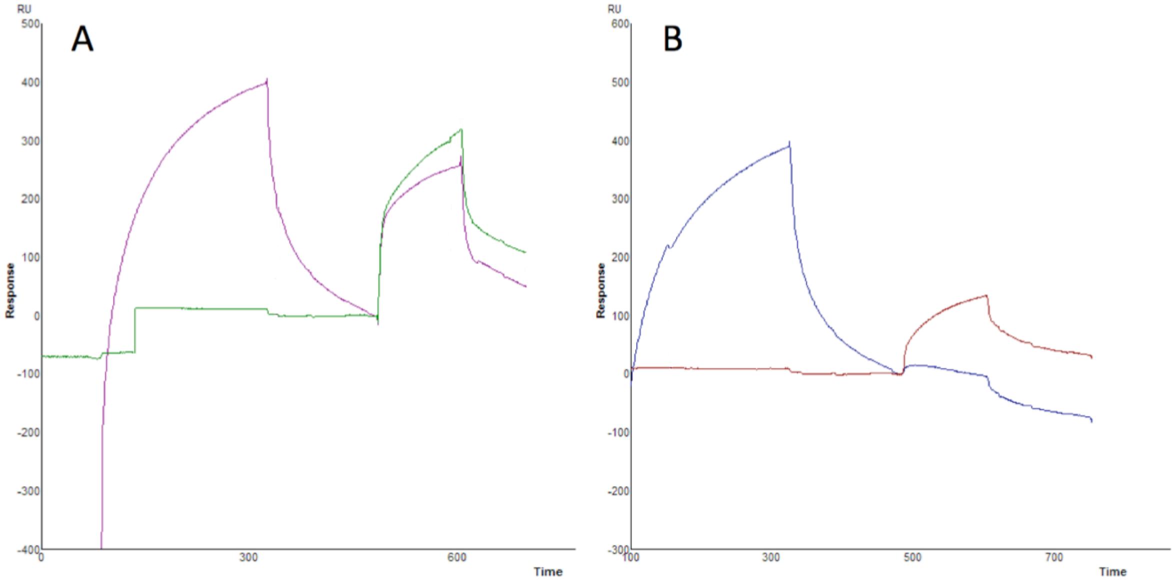

Figure S3. Biacore inhibition assays of scFv clones B13 (A) and B14 (B) to demonstrate differences in epitope recognition. In A. binding of B13 to immobilized Bet v 1 is detected after prior injection of a large amount of B14 (purple) or buffer alone (green). No inhibition could be detected when taking the baseline drift caused by dissociation of B14 into account. In B. the ability of prior injection of a large amount of B14 to block the binding of an additional injection of B14 to Bet v 1 is confirmed (blue) by comparison to the binding of B14 observed following an injection with buffer alone (brown).

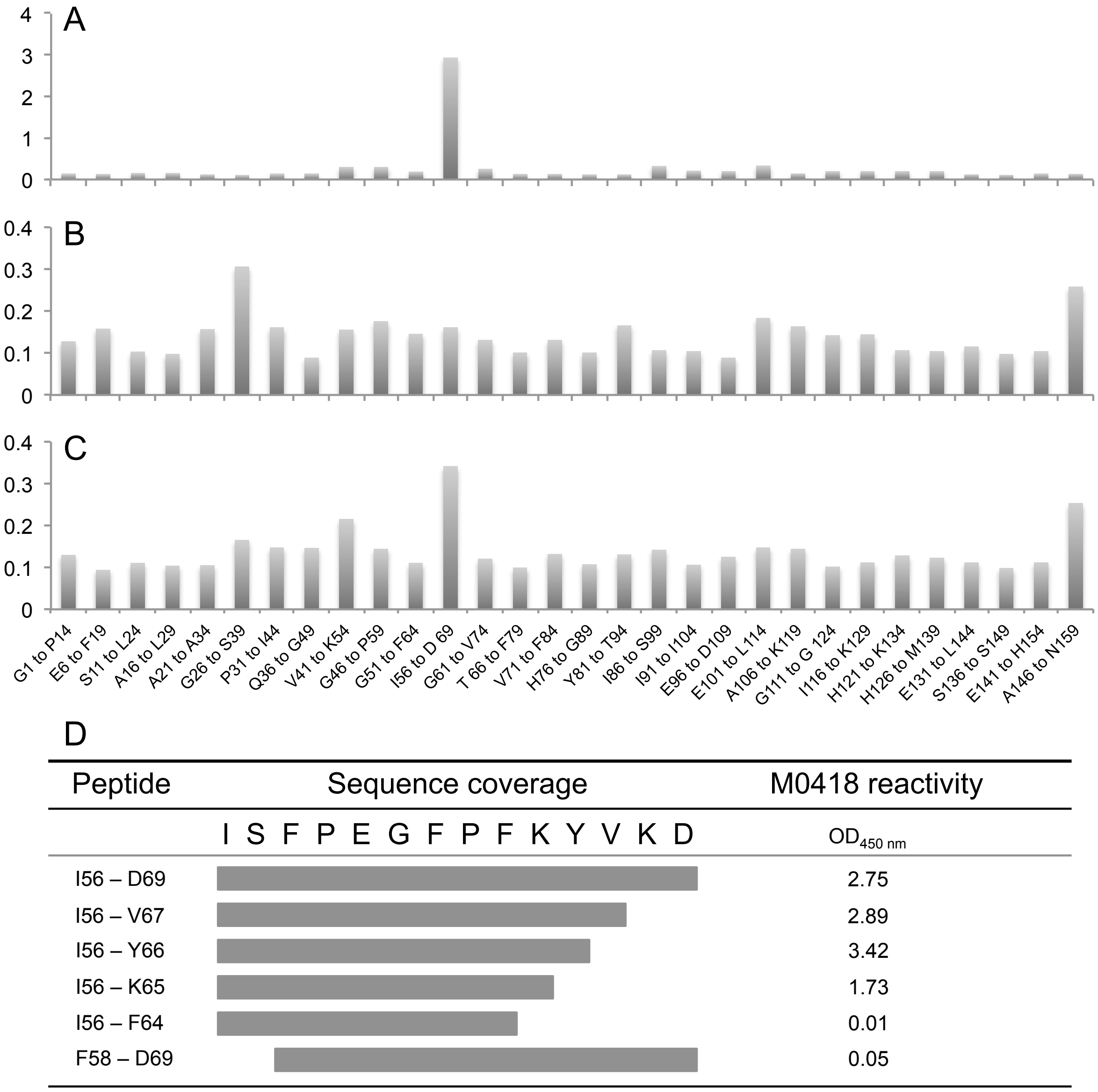

Figure S4. Epitope mapping of human monoclonal IgE M0418 (A), B13 (B) and B14 (C) (as scFv-Fcε fusion proteins) by ELISA against peptides covering the amino acid sequence of Bet v 1.0101. The identified epitope of M0418 was further pin-pointed by ELISA against shorter version of the original peptide identified (D).

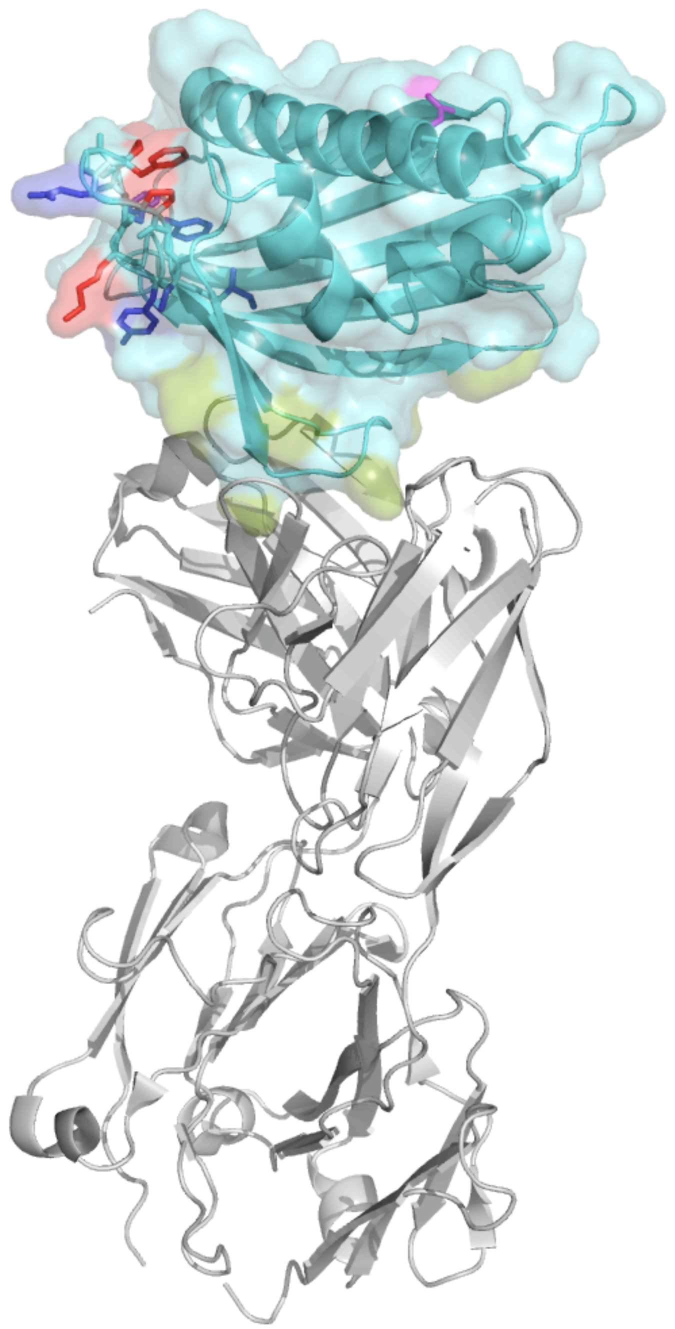

Figure S5. Mapping of epitopes on Bet v 1 defines three of the regions recognized by antibodies. One of the structures of mouse monoclonal Fab BV16 (grey; PDB: 1FSK) in complex with allergen is shown, but the Bet v 1.0112 molecule (that does not bind M0418) in the complex has been substituted with a well-aligned (R.M.S. = 0.50 Å) structure of Bet v 1.0101 (cyan; PDB: 4A80). Surfaces created by side chains of allergen residues suggested to make contact with BV16 Fab [S1] are colored in green. Side chains of allergen residues P59, F62, P63 and K65, known through peptide mapping (Figure 4) to be particularly important for the interaction between Bet v 1 and M0418, are colored in red while other side chains in the I56-Y66 sequence are colored in blue. The side chain of residue E148 in the C-terminal α–helix of Bet v 1, known to be important for the binding of another human IgE-derived scFv [S2] is highlighted in magenta. The illustration was created using PyMOL 1.3 [S3].

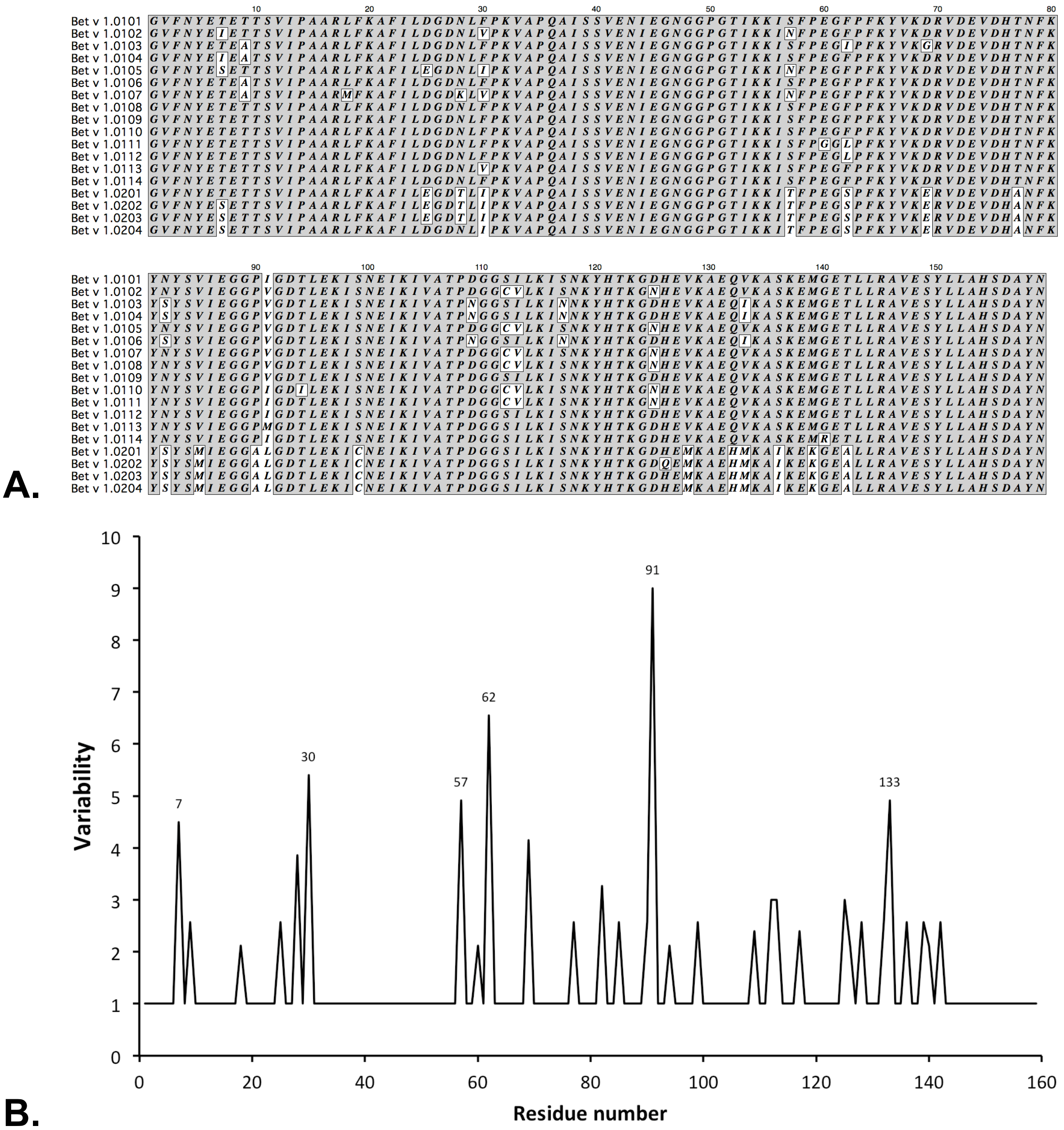

Figure S6. Sequence (A) of 18 isoallergens/isoforms of Bet v 1 and variability (B) in the different residues calculated as described by Wu and Kabat [S4]. The sequential numbers of the most diverse residues are shown.

Bet v 1.0101	I	S	F	P	E	G	F	P	F	K	Y	V	K	D
Bet v 1.0102	.	N	.	.	.	.	.	.	.	.	.	.	.	.
Bet v 1.0112	.	.	.	.	.	.	L	.	.	.	.	.	.	.
Aln g 1.0101	.	T	.	.	.	.	S	.	.	.	.	.	.	E
Car b 1.0109	.	T	.	A	.	.	S	.	.	.	F	.	.	E
Cor a 1.0101	.	T	.	G	.	.	S	R	Y	.	.	.	.	E
Mal d 1.0108	.	T	.	G	.	.	S	Q	Y	G	.	.	.	H
Que a 1.0301	.	T	.	G	.	.	S	H	L	.	H	A	.	H
Cas s 1.0101	.	T	.	G	.	A	S	K	Y	.	.	S	.	H
	*			*		*	*	*		*	*			

Figure S7. The sequence recognized by M0418 (Bet v 1 residues I56 to D69) exemplified in some members of the PR-10 family of proteins. Identical residues are shaded and indicated by a dot. Residues of Bet v 1.0101 defined by alanine scanning to be very (large asterisks) or somewhat (smaller asterisks) important for the interaction are highlighted. Note that one to four out of the four residues considered to be most important for binding differ between reactive isoforms of Bet v 1 and recombinant allergen isoforms of alder, hornbeam, hazelnut, apple, white oak and chestnut.

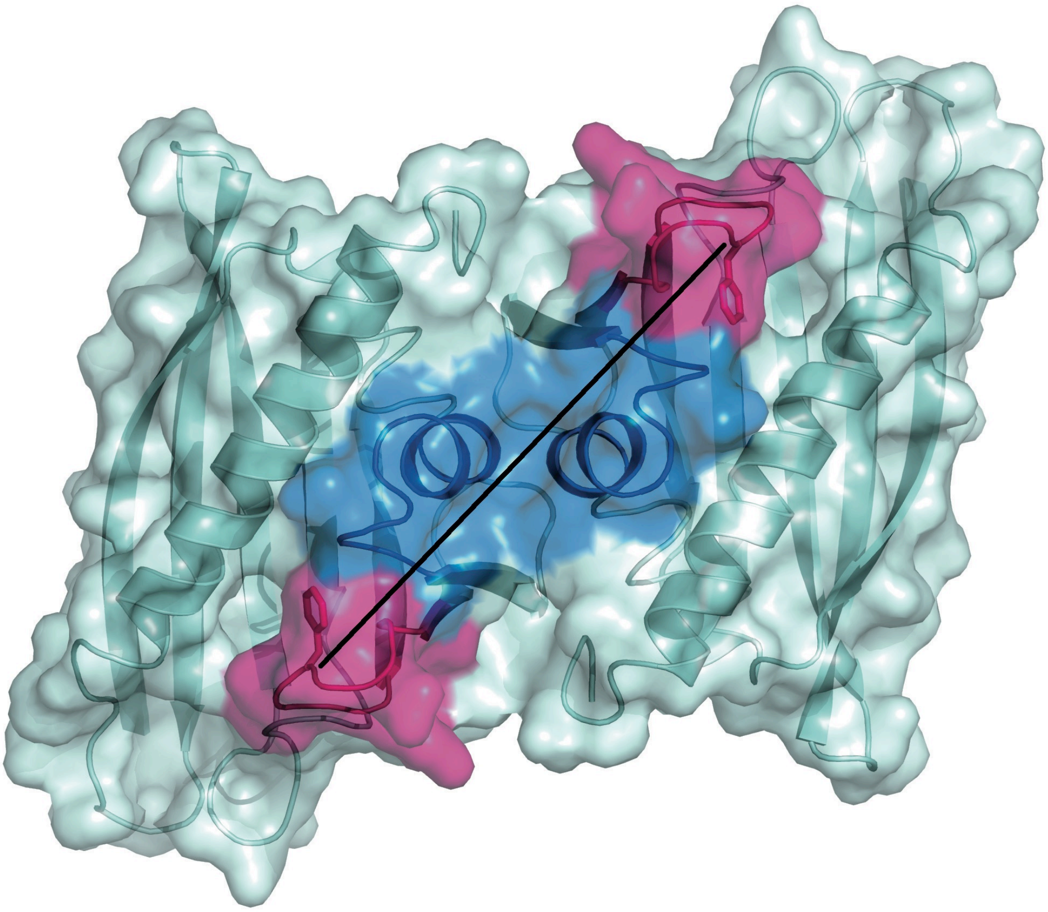

Figure S8. Proposed model of dimeric Bet v 1 [S5]. The epitope targeted by clone M0418 (residues 56-66) is colored in pink while that targeted by B13 (residues 26-39) is colored in blue. The distance between Cα atoms of Phe62 (39.6Å), a critical residue for the M0418 epitope, is indicated by a black line. In the dimeric form of Bet v 1, the M0418 epitope would be optimally oriented for FcεRI cross-linking. The figure was produced with PyMOL [S3].

Supporting Information: References

S1 Mirza O, Henriksen A, Ipsen H, *et al.* Dominant epitopes and allergic cross-reactivity: complex formation between a Fab fragment of a monoclonal murine IgE antibody and the major allergen from birch pollen Bet v 1. *J Immunol* 2000; 165:331-8.

S2 Hecker J, Diethers A, Schulz D, *et al.* An IgE epitope of Bet v 1 and fagales PR10 proteins as defined by a human monoclonal IgE. *Allergy* 2012; 67:1530–7.

S3 The PyMOL Molecular Graphics System, Version 1.3 Schrödinger, LLC.S4

S4 Wu TT, Kabat EA. An analysis of the sequences of the variable regions of Bence Jones proteins and myeloma light chains and their implicaitons for antibody complementarity. *J Exp Med* 1970; 132:211-50.

S5 Rouvinen J, Jänis J, Laukkanen M-L, Jylhä S, Niemi M, Päivinen T, Mäkinen-Kiljunen S, Haahtela T, Söderlund H, Takkinen K. Transient Dimers of Allergens. *PLoS ONE*. 2010; 5:e9037.
